# Validation and real-world clinical application of an artificial intelligence algorithm for breast cancer detection in biopsies

**DOI:** 10.1038/s41523-022-00496-w

**Published:** 2022-12-06

**Authors:** Judith Sandbank, Guillaume Bataillon, Alona Nudelman, Ira Krasnitsky, Rachel Mikulinsky, Lilach Bien, Lucie Thibault, Anat Albrecht Shach, Geraldine Sebag, Douglas P. Clark, Daphna Laifenfeld, Stuart J. Schnitt, Chaim Linhart, Manuela Vecsler, Anne Vincent-Salomon

**Affiliations:** 1 Institute of Pathology, Maccabi Healthcare Services, Rehovot, Israel; 2 Ibex Medical Analytics, Tel Aviv, Israel; 3 Diagnostic and Theranostic Medicine Division, Department of Pathology, Institut Curie, PSL University, Paris, France; 4 Department of Pathology, Shamir Medical Center, Beer Yaakov, Israel; 5 Department of Pathology, Brigham and Women’s Hospital and Harvard Medical School, Boston, MA USA; 6 Breast Oncology Program, Dana-Farber/Brigham Cancer Center, Boston, MA USA; 7 Present Address: Department of Pathology, Oncopole, Institut Claudius Regaud, Toulouse, France; 8 Present Address: GenetikaPlus, Tel Aviv, Israel

**Keywords:** Pathology, Breast cancer

## Abstract

Breast cancer is the most common malignant disease worldwide, with over 2.26 million new cases in 2020. Its diagnosis is determined by a histological review of breast biopsy specimens, which can be labor-intensive, subjective, and error-prone. Artificial Intelligence (AI)—based tools can support cancer detection and classification in breast biopsies ensuring rapid, accurate, and objective diagnosis. We present here the development, external clinical validation, and deployment in routine use of an AI-based quality control solution for breast biopsy review. The underlying AI algorithm is trained to identify 51 different types of clinical and morphological features, and it achieves very high accuracy in a large, multi-site validation study. Specifically, the area under the receiver operating characteristic curves (AUC) for the detection of invasive carcinoma and of ductal carcinoma in situ (DCIS) are 0.99 (specificity and sensitivity of 93.57 and 95.51%, respectively) and 0.98 (specificity and sensitivity of 93.79 and 93.20% respectively), respectively. The AI algorithm differentiates well between subtypes of invasive and different grades of in situ carcinomas with an AUC of 0.97 for invasive ductal carcinoma (IDC) vs. invasive lobular carcinoma (ILC) and AUC of 0.92 for DCIS high grade vs. low grade/atypical ductal hyperplasia, respectively, as well as accurately identifies stromal tumor-infiltrating lymphocytes (TILs) with an AUC of 0.965. Deployment of this AI solution as a real-time quality control solution in clinical routine leads to the identification of cancers initially missed by the reviewing pathologist, demonstrating both clinical utility and accuracy in real-world clinical application.

## Introduction

Breast cancer is the most common malignant disease worldwide, with over 2.26 million new cases in 2020^1^. Its diagnosis is determined based on the histological review of specimens acquired by needle core biopsies (NCBs), including Tru-cut biopsies for palpable masses or masses identified under mammograms, ultrasound scans, or magnetic resonance imaging and vacuum-assisted breast biopsies, especially used for sampling of calcifications, or surgical excision biopsies taken from the site of suspected malignancy. Breast cancer is classified based on morphological and cytological characteristics associated with invasive breast carcinoma. Many different histological types of breast cancers are recognized in the World Health Organization classifications^2^, the two most frequent being the invasive carcinoma of the breast of no special type (IC-NST), and infiltrating/invasive lobular carcinoma (ILC), accounting for ~70% and 10–15% of all invasive breast tumors, respectively^2^. Other invasive breast cancers, such as mucinous and metaplastic carcinomas, are significantly less prevalent^3^. Clinical management of those histological variants of invasive carcinomas are becoming different in the era of personalized medicine^4^, emphasizing the need for an accurate histological classification at initial diagnosis. Pre-invasive epithelial proliferation within the ducts encompasses ductal carcinoma in situ (DCIS) and atypical ductal hyperplasia (ADH), which are associated with significantly higher risks of subsequent invasive carcinoma, and women with these findings may require additional surveillance, prevention, or treatment to reduce their risks of developing an invasive carcinoma^3,5^. DCIS are classified as low grade (LG), medium/intermediate grade (IG), or high grade (HG), based on the morphology of the cells and their nuclei. ADH may be morphologically similar to LG DCIS, and its diagnosis will rely on the size of the lesion, or it may have a morphology that is between DCIS and florid hyperplasia^6^.

Histopathological assessment of tumor type, size, and stage of cancer, as well as phenotype, have a major impact on the choice of treatment strategies^7,8^ and are also used as prognostic aids^8,9^. Histopathological assessment of biopsies is performed using microscopic examination of tissue sections stained by hematoxylin and eosin (H&E) and by immunohistochemical techniques. Since histopathological assessments rely on pathologists’ labor, diagnostic processes may have significant limitations in the timely turnaround of pathology reports, even though breast biopsies are generally reviewed as a high priority. Moreover, the pathology workload is constantly growing due to the shortage of expert pathology workforce alongside an increase in the number of specimens with higher breast cancer incidence^10,11^ and screening programs for early cancer detection^12^. In addition, as with any manual diagnostic process, it is subject to substantial intra- and inter-reader variability, adding to the risk of misdiagnosis or overdiagnosis. The cumulative probability of a woman receiving at least one false-positive biopsy over 10 years is estimated to be between 4.8% and 9.4%^13^. An estimated 13% (1 in 8) of women in the United States will likely be diagnosed with breast cancer in their lifetime, emphasizing the need for access to high-quality diagnosis with implications on optimal treatment pathways^10^.

Automated tools for biopsy review that employ Artificial Intelligence (AI) algorithms to augment the pathologist’s effort can offer more scalable, standardized, and streamlined processes of biopsy diagnostic review and breast cancer detection, and can ultimately help optimize patient treatment. The adoption of digital imaging in pathology has grown significantly in recent years, enabling the initial deployment of AI-based tools to support routine clinical use, in particular in prostate biopsies^14,15^. For breast biopsy diagnostic support, several publications have demonstrated the feasibility of developing AI-based algorithms to support classification, with a sensitivity of 70–79%, specificity of 41–95%, and accuracy of 0.70– 0.94^16–20^ depending on the type and grade of tumors. These tools have not progressed from the academic setting into properly designed clinical studies, and none of them, to the best of our knowledge, have been deployed in a live setting in a diagnostic laboratory. Moreover, to date, none of the developed and validated AI algorithms have the capability to identify dozens of morphological features in histology samples, to the best of our knowledge.

This study presents the development of an AI algorithm with very broad capabilities for breast biopsies and the results of a blinded multi-site clinical validation assessing its performance in the detection of invasive and in situ breast carcinomas in core needle biopsies. Subsequently, an automated slide review workflow based on the developed AI algorithm is deployed as a second read (SR) application in a pathology laboratory in routine clinical practice, the first read being a microscopic or digital examination of slides by a pathologist.

## Results

### Algorithm internal testing

**Fig. 1:**
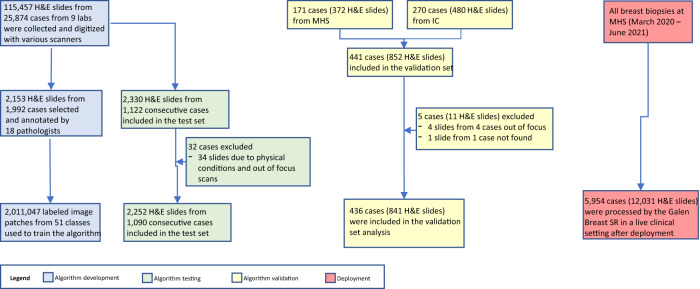
Study flow chart detailing the cases analyzed and the different study phases.

For algorithm internal testing, 2252 H&E slides from 1090 consecutive patients from Maccabi Healthcare Services (MHS) were used (Fig. 1). The mean (SD) patient age in the internal test set was 49.3 (13.9) years (Table 1). The majority of specimens were NCBs (Tru-cut) (91.4%) and the remainder vacuum-assisted biopsies (8.6%) (Table 1). Of the 1090 internal test cases, the majority were benign (80.8%), with the remaining cases presenting invasive carcinomas (15.9%) and DCIS (LG, IG, and HG)/ADH (3.3%), reflecting the real-world distribution of breast biopsies diagnoses (Table 1). Four cases of rare invasive subtypes (non-ILC and non-IC-NST) were present in the internal test set.

**Table 1:** Patient and diagnostic characteristics of the internal test and external validation sets.

Dataset		Internal test set	External validation set		
		MHS	IC	MHS	Total
Cases, *N*		1090	269 (61.7%)	167 (38.3%)	436
H&E/HES Slides, *N*		2252	479 (57.0%)	362 (43.0%)	841
Patient age at biopsy (years)					
Mean (SD)		49.3 (13.9)	52.3 (10.6)	53.5 (12.9)	52.7 (11.5)
Median		49.0	53.4	51.0	52.8
Min-Max		18-89	20-70	21-90	20-90
Patient age (years) category, *N* (%)					
<40		241 (22.1%)	37 (13.8%)	17 (10.2%)	54 (12.4%)
40–49		333 (30.6%)	70 (26.0%)	51 (30.5%)	121 (27.8%)
50–59		248 (22.8%)	84 (31.2%)	46 (27.5%)	130 (29.8%)
60–69		177 (16.2%)	77 (28.6%)	34 (20.4%)	111 (25.5%)
≥70		91 (8.3%)	1 (0.4%)	19 (11.4%)	20 (4.6%)
Biopsy type, *N*					
NCBs (tru-cut)		996 (91.4%)	176 (64.2%)	98 (35.8%)	274 (62.8%)
Vacuum-assisted/Macrobiospies		94 (8.6%)	93 (57.4%)	69 (42.6%)	162 (37.2%)
Distribution of diagnoses, *N* (%)					
Benign/others^a^		881 (80.8%)	92 (34.2%)	53 (31.7%)	145 (33.3%)
DCIS/ADH only		36 (3.3%)	76 (28.3%)	59 (35.3%)	135 (31.0%)
DCIS grades	HG/IG	23 (63.9%)	47 (61.8%)	41 (69.5%)	88 (65.2%)
	LG/ADH	12 (33.3%)	29 (38.2%)	17 (28.8%)	46 (34.1%)
	Unknown	1 (2.8%)	0 (0.0%)	1 (1.7%)	1 (0.7%)
Invasive breast carcinoma		173 (15.9%)	101 (37.5%)	55 (33.0%)	156 (35.7%)
Invasive subtype	IDC^b^	156 (90.2%)	61 (60.4%)	37 (67.3%)	98 (62.8%)
	ILC^c^	13 (7.5%)	38 (37.6%)	17 (30.9%)	55 (35.3%)
	Other^d^	4 (2.3%)	2 (2.0%)	1 (1.8%)	3 (1.9%)

*NCBs* needle core biopsies, *ADH* atypical ductal hyperplasia, *DCIS* ductal carcinoma in situ, *HG/IG/LG* high- / intermediate- /low- grade, *IDC* invasive ductal carcinoma, *IC-NST* invasive carcinoma no special type, *ILC* infiltrating/invasive lobular carcinoma, *IC* Institut Curie, *MHS* Maccabi Healthcare Services.

^a^Includes benign and other diagnoses, such as biphasic tumors (fibroadenoma and phyllodes).

^b^Includes IC-NST and rare subtypes, such as mucinous, papillary, tubular, etc.

^c^Include also pleomorphic and signet ring cell-like ILC.

^d^Includes metaplastic and tubulo-lobular carcinoma.

The performance of the AI algorithm measured on the internal test set for the detection of invasive carcinoma showed high accuracy: specificity of 98.27% (95% CI: 95.03%; 99.41%), sensitivity of 99.02% (95% CI: 98.15%;99.48%) and area under the ROC curve (AUC) of 0.998 (95% CI: 0.996;1.000). The PPV and NPV values for this outcome were also very high—95.0% and 99.7%, respectively. In addition, high performance was received when differentiating the DCIS type tumor from benign and other types of non-invasive tumors, with a specificity of 98.64% (95% CI: 97.56%;99.30%), a sensitivity of 100% (95% CI: 84.50%;100%), and AUC of 0.999 (95% CI: 0.997;1). The performance on differentiating between IDC (including IC-NST and rare subtypes) and ILC demonstrated an AUC of 0.932 (95% CI: 0.862;1.000); the number of ILC cases was too low to estimate additional statistics (Table 2).

**Table 2:** AI algorithm performance on the internal test and external validation sets.

Set	Analysis	Number of cases	AUC [95% CI]	Specificity [95% CI]	Sensitivity [95% CI]	PPV NPV
Internal test set (MHS)	Invasive vs. non-invasive	1090 (173 invasive, 917 non-invasive)	0.998 [0.996;1.000]	98.27% [95.03%;99.41%]	99.02% [98.15%;99.48%]	95.0%, 99.7%
	DCIS vs. benign/other	908^a^ (27 DCIS, 881 benign)	0.999 [0.997;1.000]	98.64% [97.56%;99.30%]	100% [84.50%;100%]	69.3%, 100%
	IDC vs. ILC	169^b^ (156 IDC, 13 ILC)	0.932 [0.862;1.000]	NA^c^	NA^c^	NA^c^
External validation set (IC + MHS)	Invasive vs. non-invasive	436 (156 invasive, 280 non-invasive)	0.990 [0.984;0.997]	93.57% [90.07%;95.90%]	95.51% [91.03%;97.81%]	89.2%, 97.4%
	DCIS vs. benign/other	248 (103 DCIS, 145 benign/other)	0.980 [0.967;0.993]	93.79% [88.63%;96.70%]	93.20% [86.63%;96.67%]	91.4%, 95.1%
	DCIS/ADH vs. benign/other	280^a^ (135 DCIS/ADH, 145 benign/other)	0.949 [0.925;0.972]	86.9% [80.44%;91.45%]	87.41% [80.67%;92.08%]	86.1%, 88.1%
	IDC vs. ILC^d^	153^b^ (98 IDC, 55 ILC)	0.973 [0.949;0.996]	92.73% [82.74%;97.14%]	92.86% [85.98%;96.50%]	95.8%, 87.9%
	DCIS HG/IG vs. LG/ADH^e^	134^f^ (88 DCIS HG/IG, 46 DCIS LG/ADH)	0.921 [0.878;0.965]	84.09% [74.92%;90.41%]	84.78% [71.46%;92.74%]	91.4%, 73.6%

*ADH* atypical ductal hyperplasia, *AUC* area under the receiver operating characteristic curve, *DCIS* ductal carcinoma in situ, *HG/IG/LG* high- / intermediate- /low- grade, *IDC* invasive ductal carcinoma, *ILC* infiltrating/invasive lobular carcinoma, *IC* Institut Curie, *MHS* Maccabi Health Services, *NPV* negative predictive value, *PPV* positive predictive value.

^a^Includes only non-invasive cases.

^b^Includes only IDC (IC-NST and rare subtypes) and ILC cases, excludes other invasive cases.

^c^Cannot be reliably estimated because the internal test set included only 13 ILC cases.

^d^IDC was considered positive and ILC negative for these analyses.

^e^DCIS HG/IG was considered positive and DCIS LG/ADH negative for these analyses.

^f^134 cases excluding 1 DCIS/ADH case with no ground truth on grading.

### Algorithm external validation

The external validation set included 841 H&E and hematoxylin-eosin-saffron (HES) slides from 436 patients from two sites - Institut Curie (IC) and MHS (Fig. 1 and Table 1). Among them, 61.7% of the cases were from the IC and the rest were from MHS. Patients’ mean (SD) age was 52.3 (10.6) years in IC and 53.5 (12.9) in MHS cohorts. The external validation set was enriched with 135 DCIS/ADH cases (31.0%) and with 156 invasive cases (35.7%), including 55 invasive lobular carcinomas (ILC) and 34 rare subtypes (tubular, mucinous, invasive papillary, encapsulated-papillary, apocrine, acinic cell, metaplastic, and tubulo-lobular invasive carcinomas) (Table 1).

**Fig. 2:**
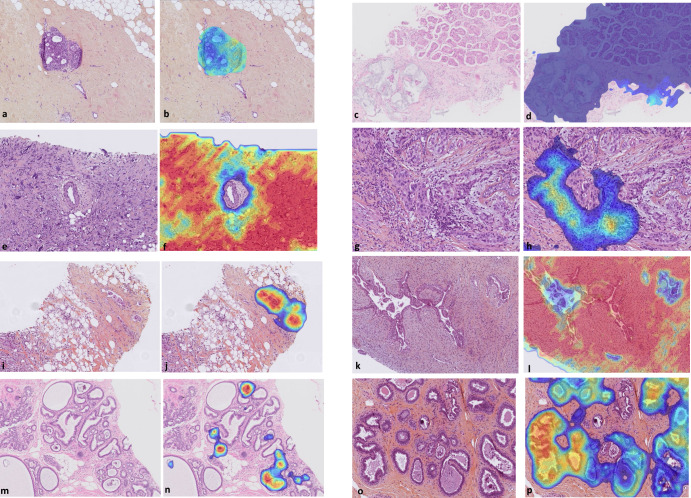
Examples of tumors and benign features identified by the AI algorithm. **a** Macrobiopsy showing atypical ductal hyperplasia focus at 8x (1.25 μm/pixel) magnification; **b** Same focus with algorithm DCIS low grade /ADH vs. DCIS high grade heatmap detecting ADH with high probability (where blue shows DCIS low grade/ADH and red shows DCIS high grade); **c** Tru-cut biopsy showing both invasive mucinous and micropapillary patterns at 8x (1.25 μm/pixel) magnification; **d** Same focus with IDC vs. ILC cancer heatmap detecting both subtypes (where blue indicates high probability for IDC and red for ILC); **e**, **f** Tru-cut biopsy with metaplastic carcinoma, detected by the algorithm with the invasive cancer heatmap at 10x (1 μm/pixel); **g**, **h** Tru-cut biopsy with IC-NST showing a focus of heavy lymphocytic infiltration, detected by the algorithm with the TILs heatmap at 16x (0.625 μm/pixel); **i**, **j** Tru-cut biopsy with IC-NST showing a focus of angiolymphatic invasion (ALI), detected by the ALI heatmap at 10x (1 μm/pixel); **k**, **l** Tru-cut biopsy with phyllodes grade I tumor, detected by the algorithm with the biphasic tumor heatmap displayed at 8x (1.25 μm/pixel); **m**, **n** Vacuum-assisted biopsy showing microcalcifications, detected by the algorithm with microcalcifications heatmap at 16x (0.625 μm/pixel); **o**, **p** Macrobiopsy with columnar cell changes, detected by the algorithm with columnar cell changes heatmap at 10x (1 μm/pixel). Slides were stained with H&E or HES. Heatmaps display low probability in blue and high probability in red, unless otherwise specified. ADH atypical ductal hyperplasia, DCIS ductal carcinoma in situ, IDC invasive ductal carcinoma, TILs tumor-infiltrating lymphocytes.

Study pathologists’ reviews based on the H&E or HES slides for ground truth (GT) determination were concordant in 378 cases (86.7%) and had discrepancies in 58 (13.3%) cases (Supplementary Table 1). Eleven (7%) cases had discrepancies in invasive diagnoses; fourteen cases (10.4%) had discrepancies between DCIS/ADH and benign diagnosis (Supplementary Table 1, see example in Fig. 2a, b). All discrepancies necessitated a third assessment by a specialist to establish ground truth. Six of the discrepancies between DCIS/ADH and benign diagnosis also necessitated a review on a multi-head microscope to reach a consensus decision, since there was no majority even after the additional reviews.

The external validation of the AI algorithm demonstrated high performance for all examined endpoints. As in the internal test results, the detection of invasive carcinoma achieved very high AUC [0.990 (95% CI: 0.984;0.997)], specificity [93.57% (95% CI: 90.07%;95.90%)] and sensitivity [95.51% (95% CI: 91.03%;97.81%)]. The PPV and NPV values for this outcome were 89.2% and 97.4%, respectively (Table 2). For the detection of DCIS, the AI algorithm demonstrated similarly high performance with an AUC of 0.980 (95% CI: 0.967;0.993), specificity of 93.79% (95% CI: 88.63%;96.70%), and sensitivity of 93.20% (95% CI:86.63%;96.67%), respectively (Table 2). The AI algorithm also demonstrated very high accuracy for the detection of the rare invasive subtypes (Supplementary Table 2), and similar performance on H&E and HES slides (Supplementary Table 3).

The AI differentiated well between subtypes and grades of invasive and in situ cancers with an AUC of 0.973 (95% CI: 0.949;0.996) for IDC (IC-NST and special subtypes) versus ILC, and an AUC of 0.921 (95% CI: 0.878;0.965) for DCIS high- and intermediate grade versus low grade/ADH (Table 2). The algorithm accurately categorized essential pathological features, including tumor subtypes, such as ILC, or rare subtypes, such as invasive mucinous, micropapillary (Fig. 2c, d), and metaplastic carcinomas (Fig. 2e, f). The AI algorithm also detected accurately tumor-infiltrating lymphocytes (TILs) (Fig. 2g, h and Supplementary Table 4), a prognostic marker in triple-negative and HER2-positive breast carcinomas, and identified angiolymphatic invasion (ALI) (Fig. 2i, j). Phyllodes tumor (Fig. 2k, l) and other pathological findings, such as microcalcifications (Fig. 2m, n) and columnar cell changes (Fig. 2o, p), were also identified by the AI algorithm and marked by heatmaps.

### Deployment in routine clinical use

Deployment of the AI algorithm as a real-time quality control system performing second reads (SR) in MHS commenced in December 2019 and is ongoing (Supplementary Fig. 1a). The system was configured to raise two types of clinical alerts—invasive cancer alert, when the AI identifies an area suspicious for invasive cancer in a case diagnosed as benign or in situ cancer; and in situ cancer alert, when the algorithm detects a focus suspicious for DCIS/ADH in a case diagnosed as benign. All slides, alerts, and other algorithmic results were accessible through the system’s web-based user interface (Supplementary Fig. 1b). The deployed system allows quick review of specific suspicious foci in a predefined percentage of the slides with the highest potential to have been misdiagnosed, which is more effective and productive than reviewing a percentage of randomly selected slides. The results presented here are for the period of March 2020 to June 2021. The system processed 5954 cases (12,031 H&E slides), including 1107 invasive carcinomas, 231 DCIS/ADH, 131 with both invasive carcinoma and DCIS, and 4485 benign cases. Invasive cancer alerts were raised for 363 (4.2%) slides from 272 cases initially diagnosed as benign, and for 136 (15.1%) slides from 81 cases initially diagnosed as DCIS/ADH. In situ cancer alerts were raised for 333 (3.8%) slides from 237 cases initially diagnosed as benign. Upon pathologist’s review of these alerts, 75% of alerts required no diagnostic amendment; most were identified as either necrosis/fat necrosis (26%), fibroadenomatous changes (23%), hyperplasia (11%) and other features (15%) (e.g., skin, hemorrhage, plasma cells) consistent with rare or irregular features that visually mimic malignant features. 25% of the alerts led to additional sections/stains being ordered, 2% of which led to an additional (third) opinion request. Alerts were focused on specific areas and visualized with associated heatmaps for invasive, IDC, ILC, DCIS, and DCIS grading, as well as other relevant features (e.g., TILs, ALI, tumor necrosis, and microcalcifications) requiring minimal pathologist review time (Supplementary Fig. 1c). The system’s performance during the deployment, was measured against the sign-out reports (that were considered GT) with an AUC = 0.990 (95% CI: 0.984;0.995), sensitivity of 98.09% (95% CI: 95.68%;99.33%), and specificity of 96.24% (95% CI: 94.70%;97.38%) for the detection of invasive carcinoma and AUC = 0.972 (95% CI: 0.949;0.996), sensitivity of 92.30% (95% CI: 83.41%;97.23%), and specificity of 92.22% (95% CI: 90.12%;93.93%) for the detection of DCIS/ADH.

### Discrepancies and misdiagnoses during external validation and deployment

**Fig. 3:**
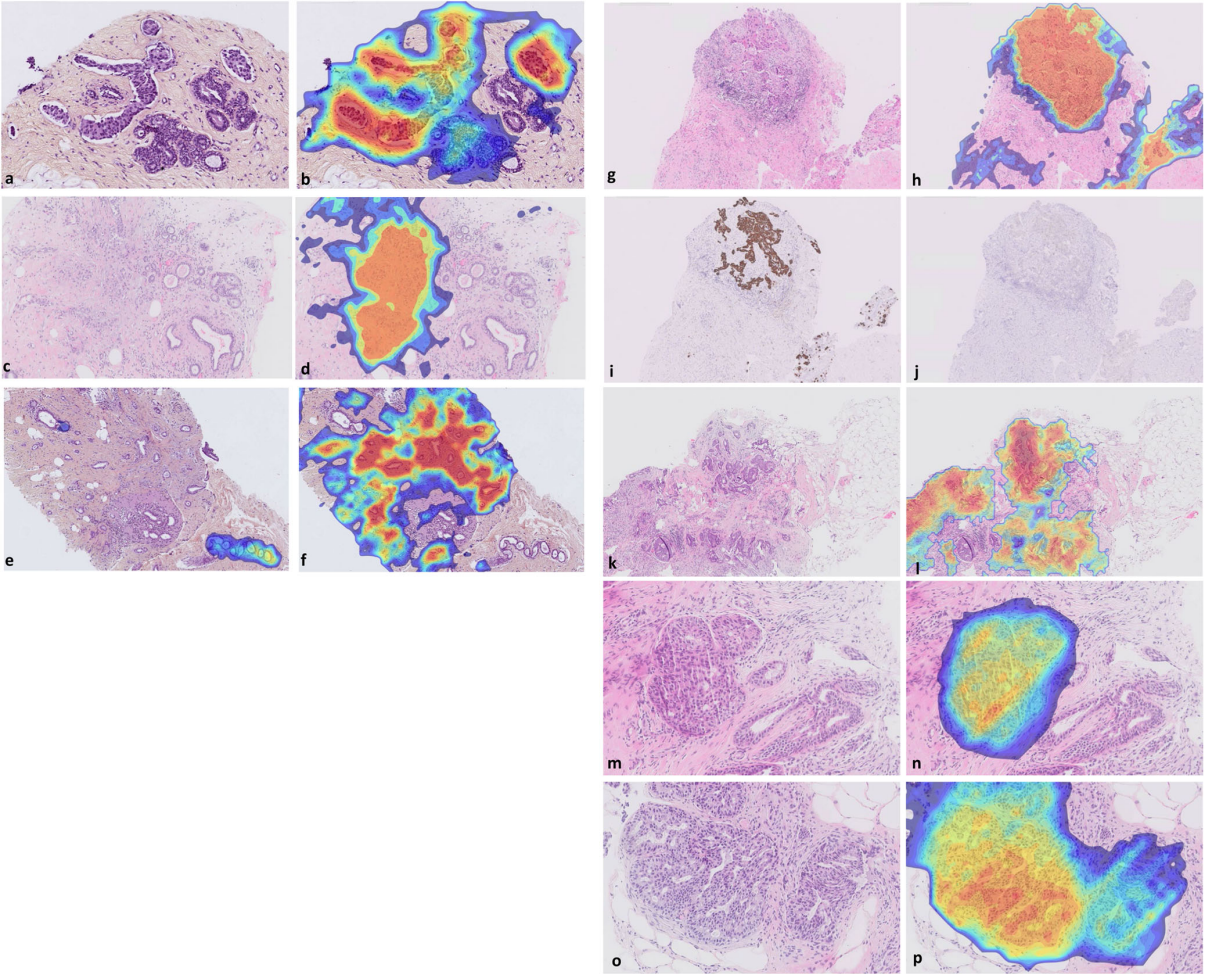
Examples of misdiagnoses identified by the algorithm. **a** Tru-cut biopsy originally diagnosed as benign with an invasive component represented only by a rare lymphovascular invasion at 16x (0.625 μm/pixel) magnification and **b** detected by the algorithm with the ALI heatmap (high probability areas in red, low probability in blue); **c**, **d** Vacuum biopsy with foreign body reaction where ILC was missed by the study pathologist, but detected by the algorithm with the invasive cancer heatmap displayed at 10x (1 μm/pixel); **e** Tubular carcinoma surrounded by fibrocystic changes at 8x (1.25 μm/pixel), detected by the algorithm (fibrocystic change heatmap) that was missed by the study pathologist; **f** Same focus detected by the algorithm with invasive cancer heatmap; **g**, **h** MRI-guided biopsy originally diagnosed as DCIS showing one of the foci of invasive cancer that were detected by the algorithm with the invasive cancer heatmap displayed at 5x (2 μm/pixel); Same focus on the IHC slides **i** positive for pancytokeratin (Ckmnf-116) and **j** negative for the myoepithelial marker (p63) confirming the diagnosis of IDC; **k**, **l** IC-NST focus missed by the original diagnosis and detected by the algorithm at 4x (2.5 μm/pixel) magnification; **m**, **n** Missed ADH case originally diagnosed as benign, alerted by the algorithm with DCIS/ADH heatmap at 16x (0.625 μm/pixel); **o**, **p** Missed ADH case originally diagnosed as benign with fibrocystic disease, alerted by the algorithm with DCIS/ADH heatmap at 16x (0.625 μm/pixel); All slides were stained with H&E or HES. Heatmaps display low probability in blue and high probability in red, unless otherwise specified. ADH atypical ductal hyperplasia, ALI angiolymphatic invasion, DCIS ductal carcinoma in situ, IC-NST infiltrating/invasive ductal carcinoma no special type, ILC infiltrating/invasive lobular carcinoma.

During algorithm external validation, eleven cases identified as invasive in the ground truth process had discrepancies between the study pathologists (Supplementary Table 1). Of these, four discrepancies were between invasive versus benign, i.e., one of the study pathologists diagnosed these as benign and the other as invasive carcinoma. All four cases were detected by the algorithm as invasive, which was their final ground truth diagnosis. One of these cases had an invasive component represented only by a rare lymphovascular invasion at one edge of the biopsy that was missed by the original reporting pathologist (Fig. 3a, b). Two cases of ILC, one with a diffuse pattern and the second associated with granulomatous mastitis with foreign body reaction, fat necrosis, and multinucleated giant cells and hemosiderin-laden macrophages (Fig. 3c, d), and one case of tubular carcinoma surrounded by fibrocystic changes and columnar cell lesions (Fig. 3e, f) were missed by a study pathologist.

During deployment, several cancer cases that had been misdiagnosed by the original pathologist were detected by the algorithm and subsequently reviewed, leading to an amended sign-out report. Here we report on four such examples. An MRI-guided biopsy of the left breast upper quadrant from a 30-year-old woman was initially diagnosed by the pathologist as DCIS high grade suspicious of microinvasion, ER and PR negative. Following an alert for the presence of invasive cancer by the AI, the pathologist re-examined the case and amended the report to infiltrating ductal carcinoma with several foci of infiltration with apocrine features and high-grade DCIS (Fig. 3g, h). These features were confirmed by IHC results, with p63(−) and CKMNF-116(+) (Fig. 3i, j). Importantly, Her2 on the infiltrative foci was scored 3+ (positive) and Ki67 was expressed in 50% of the tumor cells, with a high impact on the patient’s correct treatment pathway. Another example of a Tru-cut biopsy from a 39-year-old female patient was diagnosed by the pathologist as DCIS with microinvasion and metastasis in a lymph node. The AI raised an alert for the presence of invasive cancer with a high probability (>0.99). Subsequent re-examination by the pathologist led to a revised diagnosis of IC-NST, the size of the invasive lesion being larger than 1 mm (Fig. 3k, l). Another example was a Tru-cut biopsy, where the pathologist initially diagnosed fibroadenomatoid changes with ductal hyperplasia. Following an alert for the presence of DCIS/ADH by the AI, level sections and IHC (myosin) were ordered and examined, and the diagnosis was revised to ADH (Fig. 3m, n). A fourth example was a biopsy originally diagnosed by the pathologist as a fibrocystic disease with florid ductal hyperplasia. Following an in situ cancer alert, deeper level sections and IHC (p63) were ordered, and the pathologist revised the diagnosis to ADH and florid ductal hyperplasia (Fig. 3o, p).

## Discussion

In this work, we report on a multi-feature clinical-grade AI algorithm that detects a wide range of histologic features, beyond breast carcinomas, in whole slide images (WSI) stained with either H&E or HES and digitized by two different scanners (Philips® and Hamamatsu®). As was demonstrated in a large-scale multi-site blinded external validation study, the AI algorithm was able to identify invasive carcinoma, including multiple rare subtypes, with high accuracy, irrespective of staining protocol and scanner type. Specifically, the algorithm accurately identified subtypes of invasive carcinomas (IC-NST, ILC, and special subtypes) and in situ carcinoma and atypia (DCIS and ADH), as well as differential in situ grading (DCIS HG/IG versus LG/ADH). The algorithm also highlighted additional features, such as TILs, ALI, columnar cell changes, and microcalcifications. To the best of our knowledge, this is the first report of an AI-based algorithm that can accurately detect such a wide range of clinically significant pathological features. Moreover, this is the first-ever implementation of an AI-based tool for breast biopsies in routine clinical use in a pathology laboratory.

Several deep-learning image analysis algorithms have been developed to assess breast cancer type and grade and show performance characteristics for invasive and in situ cancer detection inferior to those shown herein^16–23^. Many of these prior publications report on algorithms limited to just one task^24^ and provide performance metrics verified mostly on small internal test datasets^21^. There are many previous publications reporting on clinical-grade highly accurate prostate cancer algorithms^14,24–26^; however, the progress of algorithms for breast cancer has been slower, probably due to the complexity of the breast tissue and multiple pathological lesions required to be detected and reported. Some of these prostate algorithms were based on weakly supervised deep learning and performed tasks of detecting cancer only (yes/no)^24^. The algorithm reported here, based on fully supervised learning, was trained to identify 51 different features, some of which are very small and not consistently reported (e.g., inflammation, microcalcifications, ALI, adenosis), and thus slide-level labels cannot be used for training as in weakly supervised AI. In addition, the expert manual annotations, although labor-intensive, enable training on various benign/normal tissue morphologies that might confuse the AI (e.g., atrophy, fat tissue, necrosis, stroma, and plasma cells).

An algorithm published by Cruz-Roa et al. was shown to identify the presence and extent of an invasive tumor with an F1 score of 75.86%, PPV of 71.62%, and NPV of 96.77%^19^, performance markedly lower than those demonstrated for the same endpoint in the current study probably due to the smaller training set, simpler model architecture and only positive (invasive)/negative classification. While Han et al. developed a classifier that can distinguish more classes (eight classes of benign and malignant breast tumors), its accuracy (93.2%) was demonstrated in a very small set of 21 cases^20^. Likewise, other recent studies reported the accuracy for the detection of invasive and in situ carcinoma on only very small sets of eight cases (10 WSIs)^27^ and 92 WSIs^28^, respectively. On a larger test set of 240 cases, Mercan et al. reported accuracy of 94% and 70% for the detection of invasive and in situ carcinomas, respectively^16^. In the current study, AUCs of 0.99 and 0.98 were achieved for the detection of invasive carcinoma and DCIS, respectively, in the 841 external validation slides. Moreover, the external validation set used in this study was enriched for difficult and rare lesions, such as special subtypes of IDC and ADH, and it consisted of multiple stain types and scanners. The accuracy of the AI on real-world data (i.e., all-comers in a single lab) is therefore expected to be even higher, as demonstrated by the extremely high performance in the internal test and deployment cohorts. Considering the overall combination of robust training methodology, algorithm architecture, and performance, the current study has demonstrated the highest levels of performance reported to date on the detection of invasive and in situ breast carcinomas (Table 3).

**Table 3:** Performance of algorithms in the detection of invasive and in situ breast carcinoma.

Study	Slides/case	Invasive carcinoma accuracy	In situ carcinoma accuracy
Cruz-Roa et al., 2017^19^	195 cancer cases (TGGA), 21 normal	F1 = 75.9%	Not available
		PPV = 71.6%	
		NPV = 96.8%	
Han et al., 2017^20^	21 cases (BreaKHis)	Accuracy = 0.93	Not available
Bejnordi et al., 2018^17^	330 cases (928 WSIs)	AUC = 0.96	Not available
Mercan et al., 2019^16^	240 cases	Accuracy = 0.94	Accuracy = 0.70
		Sens = 70%	Sens = 79%
		Spec = 95%	Spec = 41%
Sheikh et al., 2020^28^	92 WSIs (ICIAR2018)	Accuracy = 0.68	Accuracy = 0.64
		Max Sens = 96%	Max Sens = 83%
		Max Spec = 85%	Max Spec = 93%
Polónia et al., 2021^27^	152 ROIs (10 WSIs from 8 cases)	Accuracy = 0.92	Accuracy = 0.88
Current study	436 cases (841 WSIs)	AUC = 0.99	AUC = 0.98
		Sens = 95.5%	Sens = 93.2%
		Spec = 93%	Spec = 93.8%

*AUC* area under the ROC curve, *ROI* regions of interest, *WSI* whole slide image, *PPV* positive predictive value, *NPV* negative predictive value.

The multi-feature AI algorithm reported in this work detects an array of tissue features in addition to breast carcinoma-specific features. The algorithm was trained to identify 51 breast-specific cytological and morphological features, or classes, that may appear in breast biopsies, such as cancer-related classes (e.g., IC-NST, mucinous IDC, metaplastic carcinoma, ILC, TILs, ALI, ADH, and HG DCIS), other clinical features (e.g., columnar cell change, phyllodes tumor, and microcalcifications) and normal tissue structures (e.g., blood vessels, smooth muscle, and normal ducts). Other studies trained models on much fewer classes (e.g., six classes in Ho et al.^29^ or eight classes in Polόnia et al.^27^) and, therefore, could not identify certain important structures or pathologies, such as DCIS or ALI^17^. The detection of multiple features renders the AI algorithm to be more comprehensive, accurate, and explainable, and thus the system has the ability to support pathologists across a wider range of tasks, including finding and grading cancer, identifying cancer subtypes, and detecting other features, such as microcalcifications.

To ensure a robust and generalizable algorithm that can accurately identify dozens of classes, the algorithm was trained using annotations and labeling of thousands of areas in more than 2000 H&E slides conducted by a global and diverse team of 18 experienced, board-certified pathologists. Training slides were selected from a collection of more than 115,000 slides to ensure the representation of rare and small features. Moreover, the annotated slides were collected from nine different labs, each lab with its own slide preparation technique (e.g., H&E vs. HES stains) and WSI scanner. The results demonstrated in this study on a large, diverse external validation cohort that was enriched with rare and difficult cases support the conclusion of very strong generalizability and high accuracy of the AI algorithm.

The performance of the algorithm reported here was established by an extensive, rigorous, blinded external validation, which is not often the case with AI algorithm studies. The majority of studies performed cross-validation and internal algorithm testing, and very few previous studies examined a few hundred cases^16,17,19^. None of the studies included a deployment phase of the magnitude reported here —5954 cases and 12,031 slides during deployment, with the total number of patients in the internal testing, external validation, and deployment stages amounting to 7480 cases and 15,124 slides. Such rigorous, large-scale validation is crucial for AI tools that are deployed in live clinical settings to ensure their safety and applicability.

One of the most important parts of the work presented here is the deployment of the AI algorithm in a routine clinical care setting. The utility of digital tools developed for clinical routine depends in equal parts on the tools’ performance and their usability. The ability to integrate within a clinical setting and demonstrate measurable value to the existing healthcare processes is one of the strengths of our system, as was proven by the examples of missed invasive and DCIS/ADH cases that had been detected during the deployment of the algorithm as a real-time quality control system, providing 100% QC on all breast biopsies. Having an objective tool that supports pathologists in their review is particularly significant when considering the substantial intra- and inter-reader variability among the pathologists. A study that examined the inter-observer diagnostic concordance for breast biopsy diagnoses among pathologists reported only around 75% agreement, with the lowest levels of agreement seen with DCIS and atypia, as low as 48%^30^. Given these rates, an automated second read system such as the one herein can add objectivity and reproducibility to the interpretation, resulting in more consistent and accurate diagnoses. Apart from pathologists’ variability, increasing pathology workloads^31^ and a shortage of pathologists in many countries^12,32,33^ preclude performing effective quality control schemes. Specifically in the United States, a 17.53% decrease in the number of pathologists was reported from 2007 to 2017^12^, with a resulting imbalance between the increase in pathology workload and the number of available trained pathologists. Pathologist workforce concerns have been raised since the 1960s^34^, emphasizing the need for AI algorithms to provide support to pathologists and thus ensure more timely and qualitative diagnoses. This need is especially relevant considering the increase in the percentage of biopsies with invasive breast cancer diagnoses in the United States^35^.

Our study has some limitations. The AI algorithm was developed to identify pathologies in female breast biopsy specimens on H&E/HES-stained slides. In the work reported here, only biopsies, and no surgical specimens, were included. For future versions, the algorithm is also being trained on surgical specimens, including the detection of margins, Nottingham grading, and quantification of IHC breast panel biomarkers. In addition, this study was not powered for the rare findings, such as ILC pleomorphic, LCIS, rare breast cancer subtypes, etc., and ongoing studies are focused on assessing performance characteristics of additional features identified by the AI, assessment of which was beyond the scope of this study, including quantification of tumor-infiltrating lymphocytes and accuracy of microcalcifications identification, as well as studies reporting on the implementation of this algorithm in different workflows, such as first read and associated clinical utility are underway.

In summary, the AI algorithm demonstrated exceptionally higher levels of performance compared to that from published literature. The main strengths of the AI algorithm stem from the strongly supervised pathologist-based training methodology, the size and diversity of the training dataset, and the rigorous testing and validation processes that the algorithm has undergone during and after its development. Following deployment, the clinical utility of this algorithm was demonstrated by diagnoses of cases revised by pathologists following alerts for a high probability of invasive or in situ carcinoma. Accordingly, the main value introduced by our algorithm is in the minimization of diagnostic errors. Thus, the AI algorithm deployed as a second read solution allows laboratories for the first time to implement an extremely effective and accurate quality control process with a very small added work in terms of pathologist time. Such algorithms can offer an important tool for computer-aided diagnosis in routine pathology practice impacting diagnosis quality and standardization of reporting to ultimately improve patient management.

## Methods

### Study design

The study followed four key steps: (1) development of an AI algorithm for breast pathology detection in WSIs of biopsies; (2) algorithm internal testing; (3) blinded algorithm validation in an independent dataset; and (4) algorithm deployment in routine clinical use. Each of these steps is described below.

Institutional review board approval for secondary data use and waiver of informed consent were obtained for this study [MHS Ethics Helsinki Committee #0153-16-ASMC; IC Ethics Committee #DATA200210] and for the real-time quality control on de-identified patient samples [MHS Ethics Helsinki Committee #0081-18-BBL]. All study data were anonymized or de-identified, and metadata for each case was recorded.

### Algorithm development

The AI algorithm was designed to identify findings of invasive breast carcinoma, including IDC (IC-NST and rare subtypes), ILC, and other subtypes, as well as DCIS and ADH and their respective grade, and other clinical and morphological features, when applied to digitized glass slides of breast biopsies stained with H&E or with HES. The algorithm is based on multilayered convolutional neural networks (CNNs) and was specifically designed to classify and analyze a WSI in three consecutive steps (based on the previously developed algorithm for prostate biopsies^14^): tissue detection, classification, and slide-level analysis. For this study, the original algorithm [see Pantanowitz et al.^14^ for details] was updated as described below.

As in the previous study, the tissue detection step runs on patches of size 256 × 256 pixels area at magnification 5x and classifies each patch into one of three classes: tissue, blurry (out-of-focus) tissue, and background. This was achieved using a Gradient Boosting classifier that uses multiple features, such as the mean and standard deviation of each RGB channel, the mean and variance of each channel in the HSV color space, the variance of the Laplacian of the patch (used to improve the detection of blurry areas), etc. In this study, the tissue detection algorithm was extended so that it could be applied to breast WSIs. The training dataset of the original algorithm, which consisted of prostate slides, was augmented by breast slides from multiple institutes, in which background and tissue areas were marked and labeled. Specifically, features that are quite rare in prostate biopsies, such as adipose tissue, were annotated to ensure that the entire breast tissue is identified correctly by the algorithm. Overall, the three classes (tissue, blurry tissue, and background) were annotated in 110 slides with 2000 annotations and 9500 patches.

The classification step was adapted to include breast-specific histological and morphological features. The breast classification algorithm generates probabilities of an image patch to belong to each of 51 predefined breast tissue classes. These classes were defined by breast specialist pathologists to include breast tissue lesions that have clinical significance and/or a distinct morphologic appearance. The classes cover different features of benign tissue (e.g., normal ducts and lobules, stroma, nerve, blood vessel, and adipose tissue), non-cancerous findings (e.g., inflammation, stromal changes, columnar cell changes, and fibrocystic changes), atypia and hyperplasia (e.g., ductal hyperplasia, ALH, ductal cancerization, lobular neoplasm, and ADH), in situ carcinoma (DCIS per grade, with necrosis, solid, etc.), invasive carcinoma and associated features (e.g., IC-NST (IDC), ILC, rare subtypes of IDC/ILC, tumor-infiltrating lymphocytes (TILs), angiolymphatic involvement (ALI), tumor necrosis), and fibroepithelial lesions (e.g., biphasic tumors).

Feeding all the patches that contain tissue (as identified in the tissue detection step) to the classification algorithm results in 51 heatmaps, each representing the probabilities of one class along the entire slide.

The classification algorithm consists of an ensemble of three networks of different architectures: Inception V1, Inception V3, and ResNet 101. Each network combines two CNNs of the same architecture—one that works on patches at higher magnification (40x/20x/10x), which can identify cytologic features, and one for patches at lower magnification (10x/5x/2.5x), which can observe morphological characteristics. The strides of the models are selected such that they will produce heatmaps of the same size. Each of the 51 heatmaps generated by each of the three models is then averaged between the models to reach the final 51 classification heatmaps.

As evidence that this setup improves the algorithm results, we calculated the log loss of each CNN per magnification, the log loss of the combination of two networks, and the log loss of the entire ensemble (Supplementary Table 5).

The CNN models were trained using transfer learning from the models used in the prostate study. In order to fine-tune the CNNs to our specific classification task, each network was trained on image patches extracted from manual annotations on 2153 H&E slides from 1992 cases, selected from 115,457 slides (25,874 cases) (Fig. 1) from nine different labs, and digitized by multiple types of scanners. The distribution of diagnoses among the 1992 cases used for training is provided in Supplementary Table 6. In addition, standard data augmentation techniques, including rotations of 90 degrees, horizontal flips, and color augmentation (brightness with a maximum delta of 64/ 255, saturation with a factor between 0.5 and 1.5, hue with a maximum delta of 0.04, contrast with a factor between 0.5 and 1.5) were used to avoid over-fitting and to encourage invariance to differences in color due to staining. A total of 2,011,047 image patches were used to train the algorithm. Training slides for the classification algorithm were selected using the following criteria: (1) randomly selected breast cases; (2) slides representing specific uncommon features in breast slides (e.g., rare subtypes of invasive cancer); and (3) slides enriched for features with a low performance by the initial algorithm. Annotations were performed by 18 senior pathologists with a range of 10– 40 years of clinical experience. The size (e.g., number of classes, annotations, and image patches) and wide diversity (e.g., rare cancer subtypes and other tissue morphologies; multiple labs, stains, and scanners) of the dataset ensured that the CNNs could converge after training to very accurate and robust models. Following annotations and training process, the main confusions that the model exhibits were related to (1) Classes with relatively scarce data (such as special types of IDC: mucinous, adenoid cystic, papillary carcinoma, etc), (2) Classes that share morphological features with other classes (such as tumor necrosis and HG DCIS, which both contain areas with necrosis), and (3) Classes whose classification by pathologists is subjective (such as ADH).

The training of the models was performed using stochastic gradient descent with a different optimizer and learning rate schedule for each CNN model: For Inception V1 and Inception V3, we used a momentum of 0.9, and for ResNet 101, we used Nesterov momentum of 0.9. Initial learning rates varied between 0.01 and 0.0001. Training of all three models took a total of 40 h on a single RTX 2080 Ti GPU.

As in our previous algorithm^14^, during the final step of the slide-level analysis, the trained CNN-based models generated slide classification results and computed a WSI-level and case-level score. For example, to determine whether a given WSI/ case was suspicious of invasive carcinoma, the software summed up the probabilities of the invasive related classes (such as IC-NST, ILC, mucinous IDC, pleomorphic ILC, TILs, and ALI) at each location in the WSI/ case, performed localized averaging with a small sliding window, and took the highest score. A similar process was performed for in situ carcinoma with in situ-related classes. Likewise, the algorithm computed case-level scores to distinguish between invasive ductal and lobular carcinomas by combining all the classes related to IDC and comparing the total score to that of all ILC-related classes. Similarly, the DCIS grading score compared the combined score of ADH and LG DCIS to the total score of IG and HG DCIS.

The case-level score is used to perform a case-level analysis of the images: The algorithm score is compared to a predefined threshold, and slides, where the score passed the threshold, are considered positive according to the algorithm, and the rest are considered negative.

### Algorithm internal testing

The internal test dataset of breast biopsies was extracted from the archive of the Pathology Institute at MHS and consisted of all consecutive cases from a defined time period (November–December 2018). This cohort was distinct and independent from the slides that were included in the algorithm training. The slides were digitized using a Philips IntelliSite Scanner (Philips Digital Pathology Solutions, Netherlands) at 40× magnification (resolution of 0.25 μm/pixel). The internal test set included 2330 H&E-stained slides from 1122 consecutive breast biopsies (Fig. 1), of which 32 (2.9%) cases were excluded, as 34 slides from these cases could not be scanned due to poor physical condition (e.g., damaged slide, broken glass) or insufficient focus. Ground truth for the internal test dataset at the case-level was based on diagnoses from the original pathology reports. The algorithm was applied to the internal test dataset in a blinded manner and algorithmic results were compared to the ground truth following unblinding.

### Algorithm external validation

The algorithm was applied to a blinded validation set, which was independent of the data on which the algorithm was developed, trained, and tested. The diagnoses were subsequently unblinded and the results of the algorithm were compared with the ground truth defined as the pathologist’s diagnoses as described below.

#### Study period and population

The external validation study was conducted on a dataset, which consisted of histopathology slides collected as part of routine clinical practice. All cases were from the pathology departments of two medical institutions: Institut Curie (IC) (France) and Maccabi Healthcare Services (MHS) (Israel). The cases were randomly selected within a predefined study period (04.2018-09.2020 in IC and 01.2019 -12.2019 in MHS), with an enriched distribution of 1:1:1 ratio of positive-invasive- positive-DCIS/ADH -to-negative cases. In addition, the set was enriched with ILC and ADH/LG DCIS cases. Specifically, after each recruited positive, the first consecutive negative was recruited until the predefined number of positives was reached.

The examined images were digitized slides originating from breast biopsies (NCBs/Tru-cut or vacuum-assisted/macrobiopsies) stained with H&E or HES, and provided with a pathology report, completely anonymized. All slides included in the study were from female subjects ≥18 years old. Cases were excluded if the scanned slides were of inadequate technical quality (e.g., large out-of-focus areas, damaged slide), or if the slides were previously included in the algorithm’s internal test set. The glass slides were scanned at 40× magnification (resolution of 0.23–0.25 μm/pixel) with Philips IntelliSite Scanner (Philips Digital Pathology Solutions; Netherlands) at MHS and with Hamamatsu NanoZoomer 360 scanner (Hamamatsu, Japan) at IC.

#### Performance definition and calculation

Ground truth was established using the following process. Two study pathologists (GB and AN) examined the slides and the diagnostic information from the original pathology report at the respective site, validating the diagnosis for each case. In parallel, two other study pathologists (experienced breast specialist pathologists, AVS and JS, with 20–40 years of experience), who were not involved in the original diagnosis and were blinded to it during the independent validation study, examined the cases and corresponding slides to determine each case as either positive or negative for invasive ductal/lobular carcinoma, DCIS and ADH, as well as DCIS grading and other important pathological features, such as TILs, ALI, hyperplasia, adenosis, columnar cell changes, etc. Each pathologist initially reviewed only H&E/HES slides, and, if requested, was provided with immunohistochemistry slides (blinded to the date of production). Ground truth was based on the consensus agreement between both study pathologists’ diagnoses. The discrepancies between the study pathologists are presented in Supplementary Table 1. For slides on which there was a disagreement between the two, a third assessment was performed by a third experienced study pathologist (AAS or LT), blinded to both sources. Following that, the majority vote was determined as the final consensus diagnosis and used as ground truth.

The ground truth for TILs was established by the consensus between two expert pathologists on the presence of TILs. While some of the experts included TILs scoring in the percentage of the stromal area occupied by TILs as per the TIL Working Group guidelines, others only indicated the presence /absence of TILs, while considering ≥30% TILs in IDC and ≥5% in ILC as positive^36^, criteria that were used to dichotomize TILs.

Sensitivity was defined as TP/(TP + FN), and specificity was defined as TN/(TN + FP), where TP was true positives, TN was true negatives, FP was false positives, and FN was false negatives. A Receiver Operating Characteristic (ROC) curve was calculated along with the area under the curve (AUC), provided with a 95% Wald confidence interval. Negative predictive value (NPV) was defined as TN/(FN + TN) and positive predictive value (PPV) was defined as TP/(TP + FP). All performance measures were provided with exact two-sided 95% confidence intervals (95% CI).

Sensitivity, specificity, NPV, PPV, and AUC of the algorithm were computed for the following endpoints:

1. Invasive cancer (IDC, including IC-NST and other subtypes, ILC, and other invasive subtypes) versus non-invasive (i.e., DCIS, ADH, benign and other cases, such as biphasic tumors and lymphomas).
2. In situ lesions (DCIS and ADH) versus benign and others (excluding invasive).
3. IDC (including IC-NST and other subtypes) versus ILC.
4. High-grade and intermediate-grade DCIS versus low-grade DCIS and ADH.

#### Sample size

To meet the performance goal of at least 80% in sensitivity and specificity, a minimal sample size of 94 positive (invasive) cases, 94 positive (in situ) cases, and 94 negative slides was calculated, assuming 80% power and a two-sided 5% level of significance.

### Statistical analysis

Statistical analyses were performed using SAS® v9.4 (SAS Institute, Cary, NC, USA). Continuous variables were summarized using a mean and standard deviation, and categorical variables by a count and percentage. The required significance level of findings was set to 5%.

### Algorithm deployment in a routine care setting

Galen Breast, the product based on the developed algorithm, was deployed in December 2019 at the Institute of Pathology of MHS, which is the second-largest healthcare provider in Israel, with a centralized pathology lab serving around 120,000 surgical pathology cases annually. Among these, ~7000 are breast biopsy specimens. The AI algorithm was implemented as a Second Read (SR) system, i.e., as a real-time quality control solution that analyzed WSIs of breast biopsies that had been reviewed and reported by pathologists using a standard of care diagnosis in live routine practice. Supplementary Fig. 1 presents the process of biopsy review with the integrated AI algorithm. The workflow started with the scanning of slides, which were automatically exported from the Philips Image Management System to the server for processing by the AI solution. In parallel, the slides were reviewed by the pathologist, as per standard lab practice, and a pathology report was generated. The AI solution did not produce a diagnosis, but alerts were raised based on a predefined alert threshold. The algorithmic score that triggered an alert (i.e., alert threshold), was set to correspond to a specificity of 95% for both invasive carcinoma and DCIS/ADH alerts (i.e., the system was configured to raise alerts for 5% of the relevant slides), thus alerts were raised for 5% most suspicious slides. Alerts do not indicate false positives, they are equivalent to choosing 5% of cases for a second review as in a regular quality control practice, but here the AI selects the most suspicious 5% slides for review. The alerts triggered a focused pathologist review of the specific region in the slide highlighted by the alert and displayed in the system’s web-based user interface (Supplementary Fig. 1b, c), where the pathologist could view the entire slide, turn on heatmaps to examine areas that received high scores for various classes, resolve alerts, and more. Since the alerts were focused on specific suspicious areas, the review time was minimal, resulting overall in a ~1% increase in the pathologists’ time, and usually did not significantly affect the sign-out time. The solution deployed at MHS raised the following alerts: (a) Invasive cancer alerts on slides from cases originally diagnosed as benign or as DCIS/ADH that have a high probability of invasive cancer; (b) In situ lesions (DCIS/ADH) alerts on slides from cases originally diagnosed as benign that have a high suspicion of DCIS/ADH.

Overall, the implementation of the AI solution and integration in an existing workflow took 1 month. Training sessions were held for all system users, including the technicians scanning the slides.

## Supplementary information

Supplementary Information

## Data Availability

The data collected during this study for algorithm training and validation was patient data obtained under Ethical Committees’ approval and was provided to the researchers through a restricted-access agreement that prevented its sharing with a third party or publicly. Future access to the external dataset can be considered through direct application for data access. Aggregate data were available within the manuscript and its Supplementary information.
