# Supplementary material for: Validation and real-world clinical application of an artificial intelligence algorithm for breast cancer detection in biopsies
Source: NPJ Breast Cancer. 2022 Dec 6;8:129. doi: 10.1038/s41523-022-00496-w (PMC9723672; doi:10.1038/s41523-022-00496-w)
Supplement: Supplementary file 1 — Supplementary Information [file 41523_2022_496_MOESM1_ESM.pdf]

**SUPPLEMENTARY INFORMATION**

**SUPPLEMENTARY TABLES**

**Supplementary Table 1** Discrepancies between the study pathologists in the external validation set

| **Diagnosis** | **Discrepancies N (%)** | **Discrepancies details - N** |
|---|---|---|
| Invasive vs. non-invasive | 11 (7%) | Invasive vs. benign – 4<br>Invasive vs. DCIS/ADH – 7 |
| IDC vs. ILC vs. other invasive | 7 (4.5%) | IDC vs. ILC – 5<br>IDC vs. Metaplastic – 2 |
| DCIS/ADH vs. benign | 14 (10.4%) | DCIS IG/HG vs. Benign – 9<br>DCIS LG/ADH vs. Benign – 5 |
| DCIS HG/IG vs. LG/ADH | 25 (18.5%) | DCIS HG vs. LG/ADH – 10<br>DCIS IG vs. LG/ADH – 15 |

ADH – atypical ductal hyperplasia; DCIS – ductal carcinoma in-situ; HG/IG/LG – high- / intermediate- /low- grade; IC-NST– infiltrating/invasive carcinoma no special type (IDC); ILC – infiltrating/invasive lobular carcinoma.

**Supplementary Table 2.** AI algorithm performance on rare invasive subtypes detection in the external validation set

| **Set** | **Analysis** | **Number of cases** | **AUC[a,b] [95% CI]** | **Specificity [95% CI]** | **Sensitivity [95% CI]** | **PPV NPV** |
|---|---|---|---|---|---|---|
| **External validation set (IC + MHS)** | **Rare Invasive vs. non-invasive** | 314<br>34 rare invasive<br>280 non-invasive | 0.992<br>[0.985;0.999] | 94.12%<br>[79.93%;99.35%] | 95.71%<br>[92.58%;97.61%] | 72.7%<br>99.3% |

[a]AUC = area under the ROC curve

[b]The AUC of the precision-recall curve is 0.9434 and the F1 score is 87.5%

**Supplementary Table 3.** AI algorithm performance on H&E- and HES- stained biopsies of the external validation set*

| Set | Analysis | Number of cases | AUC [95% CI] | Specificity [95% CI] | Sensitivity [95% CI] | PPV NPV |
|---|---|---|---|---|---|---|
| H&E | **Invasive vs. non-invasive** | 167<br>55 invasive<br>112 non-invasive | 0.991<br>[0.981;1] | 93.75%<br>[87.10%;97.50%] | 96.36%<br>[86.40%;100%] | 88.3%<br>98.1% |
| | **DCIS vs. benign/other** [a] | 103<br>50 DCIS<br>53 benign/other | 0.998<br>[0.994;1] | 98.1%<br>[88.62%;100%] | 98%<br>[87.99%;100%] | 98%<br>98.1% |
| | **IDC vs. ILC** [b,c] | 54<br>37 IDC<br>17 ILC | 0.976<br>[0.944;1] | 94.12%<br>[69.24%;100%] | 91.9%<br>[76.98%;98.93%] | 97.1%<br>84.2% |
| | **DCIS HG/IG vs. LG/ADH**[d] | 58<br>41 DCIS HG/IG<br>17 DCIS LG/ADH | 0.937<br>[0.879;0.995] | 82.35%<br>[55.80%;96.98%] | 82.92%<br>[67.40%;92.86%] | 91.9%<br>66.7% |
| HES | **Invasive vs. non-invasive** | 269<br>101 DCIS/ADH<br>168 benign/other | 0.989<br>[0.980;1] | 93.45%<br>[88.29%;96.67%] | 95.05%<br>[88.28%;98.52%] | 89.7%<br>96.9% |
| | **DCIS vs. benign/other** [a] | 145<br>53 DCIS<br>93 benign/other | 0.960<br>[0.931;0.988] | 89.13%<br>[80.50%;94.63%] | 88.68%<br>[76.28%;95.85%] | 82.5%,<br>93.2% |
| | **IDC vs. ILC** [b,c] | 99<br>61 IDC<br>38 ILC | 0.971<br>[0.938;1] | 92.11%<br>[77.52%;98.96%] | 91.8%<br>[81.17%;97.48%] | 94.9%,<br>87.5% |
| | **DCIS HG/IG vs. LG/ADH** [d] | 76<br>47 DCIS HG/IG<br>29 DCIS LG/ADH | 0.913<br>[0.852; 0.975] | 82.75%<br>[63.51%;94.34%] | 82.98%<br>[68.65%; 92.32%] | 88.6%<br>75% |

*The small differences between the two sets could stem from differences between the scanners and/or between the stains (H&E vs HES) and/or between the labs (different preprocessing of tissue).

[a] Includes only non-invasive cases; [b] Includes only IDC (IC-NST and rare subtypes) and ILC cases, exclude other invasive cases; [c]IDC was considered positive and ILC negative for these analyses; [d]DCIS HG/IG considered positive and DCIS LG/ADH negative for these analyses;

ADH – atypical ductal hyperplasia; AUC – area under the receiver operating characteristic curve; DCIS – ductal carcinoma in-situ; HG/IG/LG – high- / intermediate- /low- grade; IDC – invasive ductal carcinoma; ILC – infiltrating/invasive lobular carcinoma; NPV – negative predicting value; PPV – positive predicting value.

**Supplementary Table 4.** AI algorithm performance on TILs detection in the external validation set

| Set | Analysis | Number of cases | AUC<br>[95% CI] | Specificity<br>[95% CI] | Sensitivity<br>[95% CI] |
|---|---|---|---|---|---|
| **External validation set (IC + MHS)** | **TILs in Invasive+DCIS** | 109<br>32 TILs positive<br>77 TILs negative | 0.965<br>[0.934;0.996] | 85.7%<br>[76.3%;92%] | 93.8%<br>[78.8%;99.3%] |
| | **TILs in Invasive** | 47<br>19 TILs positive<br>28 TILs negative | 0.953<br>[0.892;1] | 85.7%<br>[67.9%;94.9%] | 94.7%<br>[73.5%;99.9%] |
| | **TILs in DCIS** | 62<br>13 TILs positive<br>49 TILs negative | 0.962<br>[0.920;1] | 85.7%<br>[73%;93.2%] | 92.3%<br>[64.6%;100%] |

**Supplementary Table 5.** Algorithm optimization process

| **Architecture** | **Inception V1** | | **Inception V3** | | **ResNet 101** | |
|---|---|---|---|---|---|---|
| Magnification | 10x | 2.5x | 40x | 5x | 20x | 10x |
| Log loss | 1.901 | 2.223 | 2.059 | 1.801 | 1.862 | 1.762 |
| Log loss of combination | 1.703 | | 1.6 | | 1.651 | |
| Ensemble log loss | 1.476 | | | | | |

**Supplementary Table 6.** Distribution of cases used for algorithm training

| **Diagnosis** | **Sub-type** | **Total Cases (Training set)** |
|---|---|---|
| Benign/Other | | 935 |
| Invasive | IDC | 391 |
| | ILC | 109 |
| | IDC+ILC | 35 |
| | Invasive Other | 42 |
| DCIS/ADH | DCIS | 266 |
| | ADH | 214 |
| **Total** | | **1992** |

DCIS – ductal carcinoma in-situ; ADH – atypical ductal hyperplasia; IDC - includes invasive ductal carcinoma (IC-NST and rare subtypes); ILC – infiltrating/invasive lobular carcinoma.

**SUPPLEMENTARY FIGURES**

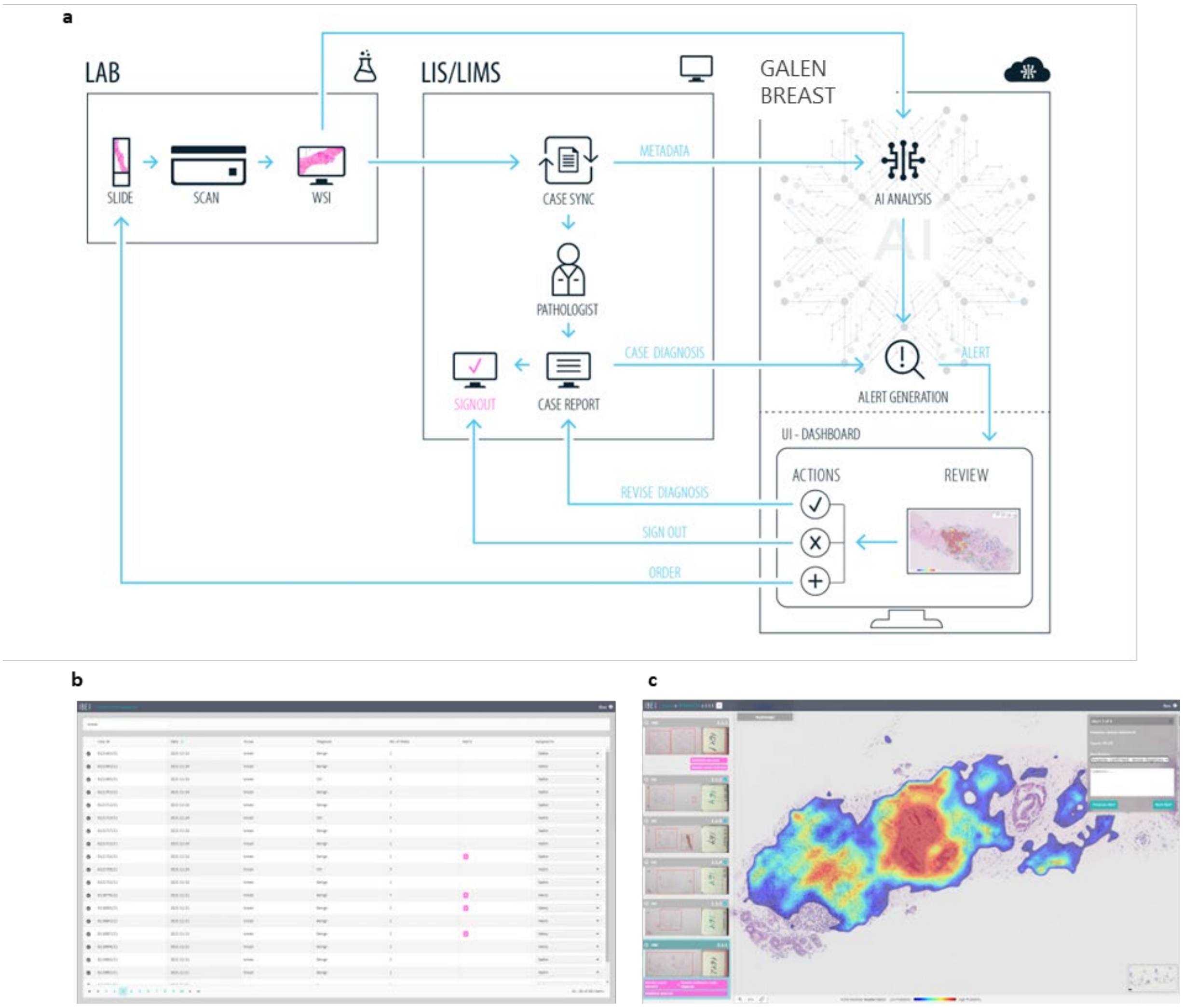


**Supplementary Figure 1**. Pathology workflow with the integrated AI-based Second Read solution.

**a.** The AI algorithm analyzed WSIs that had been diagnosed by pathologists in their routine workflow. The system raised alerts for cases with a potential discrepancy between the pathologist's diagnosis and the algorithmic results on the existence of invasive or in-situ breast cancer. Pathologists used the system's web-based user interface to review the cases; **b.** Example of a case list with pink-labeled alerts; **c.** Example of a resolved alert after the pathologist performed a second review, specifically focused on the region that triggered the alert, as highlighted by heatmap in the slide viewer.

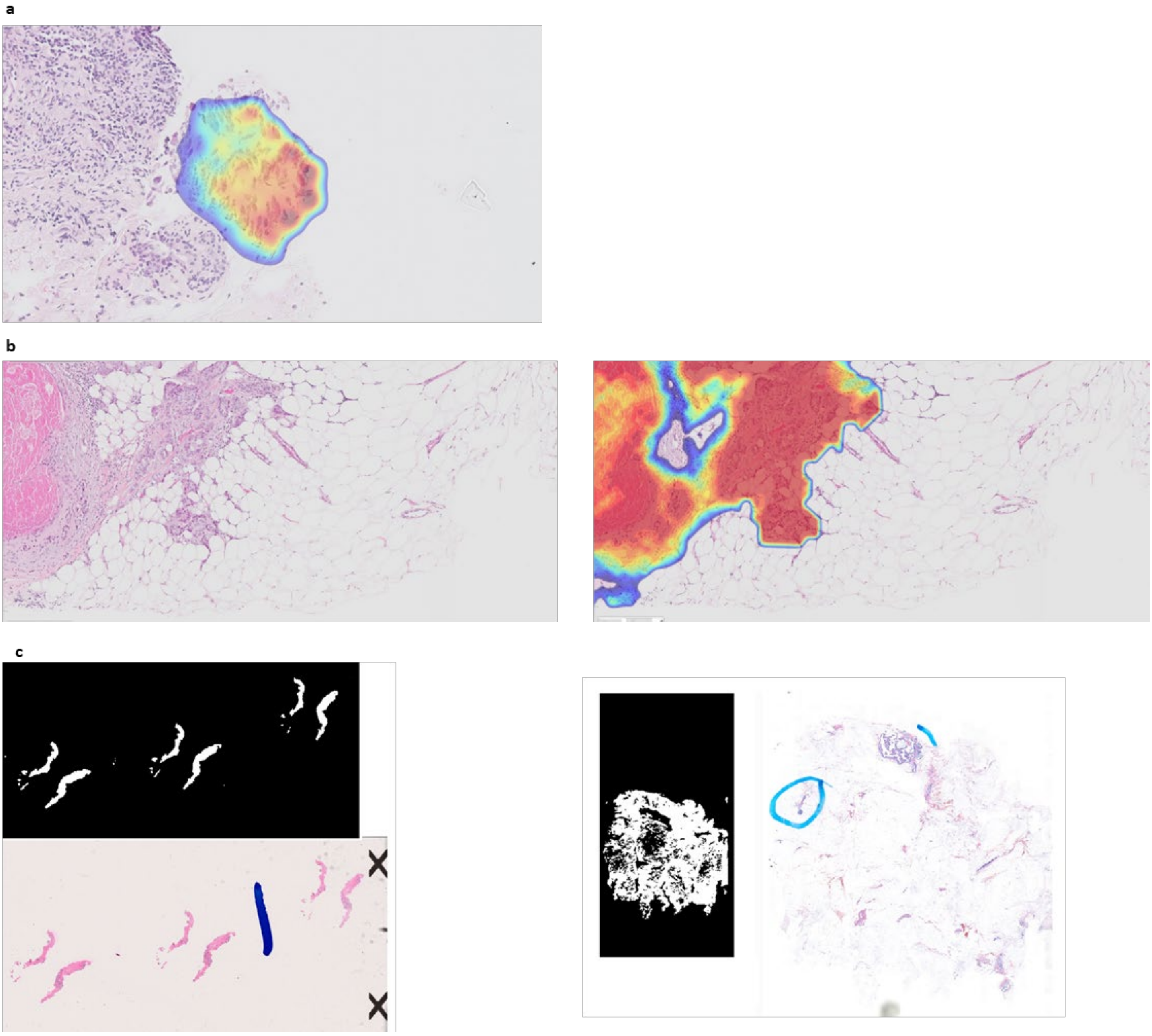


**Supplementary Figure 2** Examples of algorithm detection for problematic areas.

**a.** An image of a macrocalcification area correctly identified on the edge of a biopsy; **b.** An image of an invasive tumor area correctly detected inside a region of fat tissue (H&E --left, with invasive cancer heatmap -right, x8 magnification); **c.** Images of slides containing artifacts such as pen and bubbles and the respective tissue detection mask that indicates that these artifacts were correctly ignored by the algorithm.
